# Fast, easy and efficient: site-specific insertion of transgenes into Enterobacterial chromosomes using Tn*7 *without need for selection of the insertion event

**DOI:** 10.1186/1471-2180-6-39

**Published:** 2006-04-28

**Authors:** Gregory J McKenzie, Nancy L Craig

**Affiliations:** 1 Howard Hughes Medical Institute, Department of Molecular Biology and Genetics, Johns Hopkins University School of Medicine, Baltimore, Maryland 21205, USA

## Abstract

**Background:**

Inserting transgenes into bacterial chromosomes is generally quite involved, requiring a selection for cells carrying the insertion, usually for drug-resistance, or multiple cumbersome manipulations, or both. Several approaches use phage λ *red *recombination, which allows for the possibility of mutagenesis of the transgene during a PCR step.

**Results:**

We present a simple, rapid and highly efficient method for transgene insertion into the chromosome of *Escherichia coli*, Salmonella or Shigella at a benign chromosomal site using the site-specific recombination machinery of the transposon Tn*7*. This method requires very few manipulations. The transgene is cloned into a temperature-sensitive delivery plasmid and transformed into bacterial cells. Growth at the permissive temperature with induction of the recombination machinery leads to transgene insertion, and subsequent growth at the nonpermissive temperature cures the delivery plasmid. Transgene insertion is highly site-specific, generating insertions solely at the Tn*7 *attachment site and so efficient that it is not necessary to select for the insertion.

**Conclusion:**

This method is more efficient and straightforward than other techniques for transgene insertion available for *E. coli *and related bacteria, making moving transgenes from plasmids to a chromosomal location a simple matter. The non-requirement for selection is particularly well suited for use in development of unmarked strains for environmental release, such as live-vector vaccine strains, and also for promoter-fusion studies, and experiments in which every bacterial cell must express a transgene construct.

## Background

Expression of transgenes in bacteria is routinely accomplished by expressing the gene from an extrachromosomal plasmid. However, chromosomal expression, with its single-copy nature and inherent stability, is preferable in many situations. For example, in experiments where physiological levels of protein are desired, including genetic complementation experiments, even low copy number plasmids can yield non-physiological levels of expression making single-copy expression a better choice [1]. In addition, interpretation of experiments involving plasmids can often be confounded by the heterogeneity of the population arising from plasmid loss due to the imperfect segregation of most plasmids [2]. Recent experiments in Salmonella have shown that merely having a plasmid present in a strain can have an effect on virulence [3]. An additional difficulty with plasmids is that a selection is required to maintain them during growth. Typically this is provided by a drug-resistance (drug^R^) marker, however drug^R ^markers are undesirable in some contexts such as when bacteria will be introduced into humans (*e.g*., for live vaccine purposes [2]). Expression of transgenes from the chromosome bypasses the sub-ideal features of plasmid-based expression. However, most systems for transgene insertion are somewhat cumbersome, involving multiple steps, and also require a drug^R ^marker to allow selection for relatively rare integration events [4,5]. Those that do not involve drug selection rely on other selection strategies that involve multiple steps [6]. We describe a system that makes chromosomal transgene expression, with all its advantages, and without need of a selection, so simple to achieve that it can be routinely used.

We have developed a system for chromosomal transgene insertion that does not require a selection for transgene insertion based on the site-specific recombination system of the bacterial transposon, Tn*7*. Tn*7 *uses five proteins for transposition, TnsABCDE [7]. TnsA and TnsB form the transposase that recognizes approximately 240 bp sequences at the ends of Tn*7 *and does the breakage and joining reactions of transposition, moving the transposon into the new site. TnsD and TnsE are alternative site selectors that determine where the transposon will insert, and TnsC links TnsA and TnsB to the site selectors, activating transposition through regulation of the ATP-bound state of TnsC. TnsE mediates transposition into conjugative plasmids, whereas TnsD promotes site-specific transposition into a chromosomal "safe haven" attachment site (*attTn7*) at the 3' end of the *glmS *gene. TnsD recognizes a site in the highly conserved *glmS *gene (essential in *E. coli *[8] and likely essential in nearly all bacteria), and inserts immediately downstream of *glmS *into *attTn7*, such that no gene is disrupted, and at no discernable fitness cost to the host [7]. In cells expressing TnsABCD, transposition occurs exclusively into *attTn7*, and not into other locations [7]. Tn*7 *appears to have a broad host range, having been demonstrated to function in many species of bacteria [9].

Our overall strategy is as follows: *tnsABCD *and a transgene flanked by recognition sites for the tranposase are delivered into cells on a temperature-sensitive plasmid that can later be cured. We use transformation (or conjugation) to establish a culture of plasmid-bearing cells, induce expression of the TnsABCD proteins to promote transposition into the *attTn7 *site, and then cure the plasmid. Using this strategy the majority of cells treated acquire a transgene insertion at *attTn7*, as detailed below.

## Results

**Figure 1:**
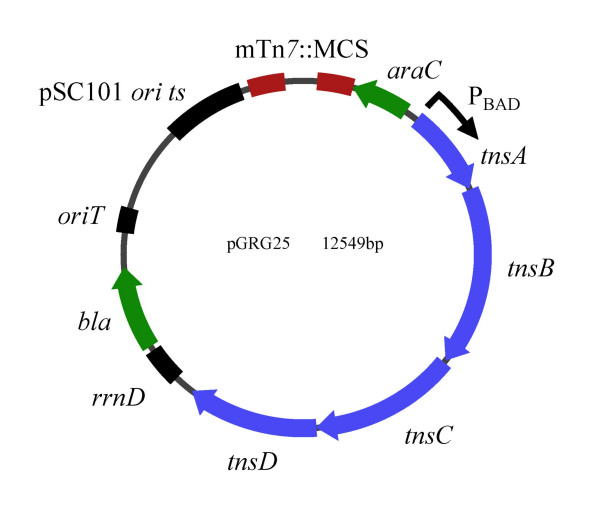
**Transposition vector**. The vector pGRG25 contains a multiple cloning site (MCS) with unique restriction sites for *Avr*II, *Not*I, *Pac*I, and *Xho*I, flanked by the terminal repeats of Tn*7*. Cloning of the gene of interest into these sites, in between the Tn*7 *arms, will result in a plasmid that can be used to insert the transgene into the chromosome. *araC *is the regulator of the arabinose-inducible promoter P_BAD _(though in all experiments presented, leaky expression was sufficient). *tnsABCD *are the genes required for transposition of Tn*7*. *rrnD *is the transcription terminator. *bla *encodes β-lactamase which allows selection of ampicillin resistance when transforming the plasmid into bacteria. *oriT *is the RP4 conjugal transfer origin. pSC101 *ori ts *is the temperature sensitive origin of replication which functions at 32°C, but not at 42°C. Vector pGRG36 is identical except for the addition of a *Sma*I site to the MCS. The sequence of pGRG25 and pGRG36 can be accessed at GenBank (accession # DQ460223).

Our integration system consists of a plasmid (pGRG25, Figure 1) carrying two key components: a multiple cloning site for transgene insertion flanked by the left and right ends of Tn*7*, and the *tnsABCD *genes expressed under control of the P_BAD _promoter [10]. *tnsABCD *are outside the transposon ends, so that they will not be inserted into the chromosome during transposition. The plasmid carries *araC *to mediate arabinose-inducible expression of *tnsABCD *genes, and a temperature-sensitive origin from pSC101 [11] to allow curing of the plasmid after transgene insertion. It also has a *bla *gene for plasmid selection, and an origin of transfer, *oriT*, to allow delivery by conjugation.

**Figure 2:**
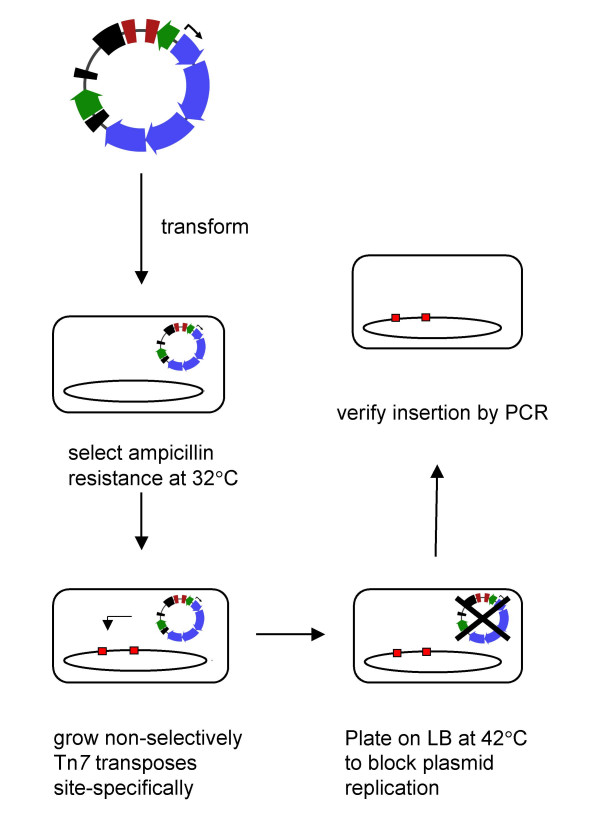
**Protocol for use of transgene insertion plasmid**. 1) Transform the plasmid into the strain of choice and select transformants on LB + ampicillin at 32°C. Introduction of the plasmid by conjugation is also an option. 2) Streak once to ensure that the cells are carrying the plasmid, then grow non-selectively in LB at 32°C. In all strains tested thus far, leaky expression of TnsABCD during this step has been sufficient to allow site-specific transposition into the *attTn7*, but 0.1% arabinose may be added to increase levels of expression of TnsABCD. 3) Plate cells at 42°C to block replication of the plasmid. 4) Streak once at 42°C to ensure loss of the plasmid, then verify insertions in the attachment site by PCR.

Transgene insertions are generated as follows (Figure 2): first a transgene is cloned into the multiple cloning site. Then, the plasmid is introduced into cells by transformation or conjugation, and a population of cells carrying the plasmid is established by selecting ampicillin resistance at 32°C on LB solid medium. These cells are then grown non-selectively in LB medium. Some bacteria may require arabinose induction of TnsABCD during this growth step, but in *E. coli *leaky expression without induction by arabinose is sufficient. When the cultures are saturated, they are plated on LB medium at 42°C for individual colonies that are then screened for presence of the transgene by PCR across *attTn7*. Table 1 shows that the majority (79%) of colonies so isolated carry the transgene insertion at *attTn7*. All colonies consisted of ampicillin-sensitive cells, indicating that the plasmid was lost. Thus, the frequency of insertion is high enough that a selection is not required to identify insertions.

Recombination mediated by TnsABCD is well documented to yield Tn*7 *insertion only at *attTn7 *[7], suggesting that in this system cells should not acquire a transgene at sites other than *attTn7*. However, to confirm this, we assayed for insertion events at other sites as follows. We used a chloramphenicol-resistant (Chl^R^) transgene to allow selection for all insertion events and selected Chl^R ^cells directly. We find that, after the procedure described above, 79 ± 8% of cells in a culture are Chl^R ^(Table 2). This is comparable to the frequency of Tn*7 *insertion into *attTn7 *(determined by PCR) when no selection is applied (Table 1). All Chl^R ^colonies tested (9/9) carry the mini-Tn*7 *at the attachment site (determined by PCR, data not shown). We conclude that TnsABCD are delivering the Tn7 specifically to *attTn7*, as expected from the many studies of Tn*7 *site-specific recombination, though our frequency of 79% is a much higher frequency than the 10^-2 ^reported previously using other *in vivo *Tn*7 *transposition assays [12].

Although the above experiment confirms that insertion is specific to *attTn7*, we wanted to further ensure that this system was not promoting insertions at other sites, given the extraordinary frequency of transposon insertion observed. To detect rare non-*attTn7 *insertion events, we made use of a property of Tn*7 *known as transposition immunity whereby Tn*7 *blocks transposition into a site already occupied by a Tn*7 *[13]. We compared the ability of our system to deliver a Chl^R ^transgene into a pair of strains, one with Tn*7 *already in the attachment site (to measure non-specific transposition frequencies) and one without Tn*7 *(to measure integration frequencies at *attTn7*). Selecting for the Chl^R ^transgene, we found that the frequency of insertion (Chl^R ^cells) was nearly 10^4 ^fold lower in the strain with a blocked attachment site (Table 2). Thus insertion at secondary sites can occur but the frequency is so much lower that it is extremely unlikely to recover a secondary non-site-specific insertion (approximately 1/10^4 ^isolated colonies will carry an insertion at a site other than *attTn7*).

## Discussion & conclusion

This system for creating chromosomal transgenes is fast, easy and efficient. Transposition occurs in 79% of cells treated (Table 1), and occurs very specifically into the Tn*7 *attachment site. This is the highest Tn*7 *transposition seen in any *in vivo *system [12], with frequencies of closer to 1% being more typical. In our system, transposition occurs at such high frequencies that selection for transgene insertion is not required. This is not the first system to employ Tn*7 *for transgene integration. However, previously described systems utilize multiple plasmids and are not efficient enough to allow detection of insertion events in the absence of a selection [14-16]. Non-specific transposition occurs at a frequency of 6.8 × 10^-5 ^in our system (Table 2), indicating that there is little worry of non-specific or secondary transposition events. We have demonstrated the utility of this system in ten members of the *E. coli *reference collection of environmental isolates (data not shown) [17] and others have successfully inserted transgenes into Enteropathogenic and Enterohemorrhagic *E. coli *isolates (R. Keller, J.W. Yoon and J. Kaper, personal communication). We have also demonstrated the utility of this system in another Enterobacterial species, *Salmonella typhimurium *(data not shown) indicating the broad utility of our approach. Our transgene integration method can be used in any bacterium that supports replication of the pSC101 origin (*i.e*., members of the *E. coli*, Salmonella, or Shigella families).

This transgene integration system accommodates large transgenes, as we have seen similar frequencies of insertion with inserts as large as 3.2 kb (data not shown). Others have used pGRG25 to insert the 7.1 kb *luxCDABE *operon into Extraintestinal pathogenic *E. coli *and observed similar insertion frequencies (P. Germon, personal communication). It is likely to be possible to insert even larger transgenes, as wildtype Tn*7 *carries 14 kb between the left and right ends.

Because the Tn*7 *attachment site is highly conserved amongst diverse bacterial species [18] this system may be useful in many different bacteria. Possible limitations of the system with regard to whether it will work in other organisms are: 1) the ability to deliver DNA *via *transformation or conjugation, 2) the ability of the plasmid to be replicated sufficiently to support transposase expression, and 3) the activity of the *araBAD *promoter driving transposase expression. Restriction of foreign DNA can have a dramatic negative effect on transformation efficiency, however in cases where restriction may be a problem, for example, with environmental isolates, the Ocr type 1 restriction inhibitor [19] (Epicentre Biotechnologies) can greatly improve transformation efficiency. Systems utilizing broad host range plasmids have been described that use Tn*7 *transposition functions to insert transgenes into the chromosome (*e.g*. [14]) in numerous different bacteria. Although these other systems work in diverse bacteria and are very useful tools for that reason, they have more components and are several orders of magnitude less efficient requiring drug^R ^markers to detect transgene insertions. The drug^R ^marker can be removed after insertion with additional steps, but clearly the system we describe here for single-step insertion without selection is simpler and easier in the organisms where we have demonstrated functionality (*E.coli*/Salmonella/Shigella).

Expanding the utility of our system to include bacteria outside the Enterobacteriaceae might be possible by incorporating a broad host range replicon combined with a negative selection for loss of the plasmid. Indeed, Tn*7 *seems the ideal molecular machine for delivering transgenes given that the *attTn7 *sequence appears to be present in most genomes examined (it occurs twice in some, but this can be dealt with as in [14]). Tn*7 *has been demonstrated to move in a broad variety of Gram negative bacteria [9] and the presence of Tn*7*-like sequences in *Bacillus cereus *and *Clostridium thermocellum *suggests that it would transpose in Gram positives as well ([20] and GenBank accession #NZ_AABG04000054).

A commonly used method for transgene insertion available prior to this work [4] is based on phage λ *red *recombination. It usually requires PCR amplification of the substrate, transformation of the *red gam *expression system and transformation of the recombination substrate, followed by curing and PCR verification. Removal of the necessary drug^R ^marker requires an additional transformation and curing. It works at frequencies at or below 10^-6^, meaning that a selection is required. The Tn*7*-based method described here inserts genes at such high frequencies that selection is not required. It requires very few steps: cloning the transgene, transformation into the strain of interest and growth at 2 temperatures, followed by PCR verification of insertion. Clearly our system is an improved method with fewer steps and greatly improved efficiency.

We anticipate this system to be useful for studies requiring single-copy gene expression, for example genetic studies, including complementation experiments. In addition, we envision that this system will be useful for development of bacterial strains for environmental release (that may not have antibiotic-resistance markers), including live-vector vaccine strains [2]. Expression of heterologous antigens in vaccine strains is currently largely plasmid-based, one reason being that plasmid copy number facilitates expression of sufficient antigen to engender an immune response. However, recent work has demonstrated that immunologically relevant levels of antigen can be expressed from the chromosome [21]. The construction of stable and selection-free chromosomal transgenes using this Tn*7*-based system should support the development of chromosomal antigen expression technology. It is worth noting that because Tn*7 *recognizes an *attTn7 *site in the human genome [18] it may be possible to create vectors for use in human cells to deliver genes at high-frequency into an innocuous site in the human genome for gene therapy.

## Methods

### Vector construction

Plasmid pCW4 [22], containing *tnsABCD*, was digested with *Pac*I and partially digested with *Drd*I, to liberate a 6636 bp *Pac*I-*Drd*I fragment. The overhanging ends were made blunt with Klenow fragment and ligated to *Sma*I linearized pBAD18 [10]. The plasmid containing *tnsABCD *in the correct orientation relative to the P_BAD _promoter was named pGRG2. pGRG2 and pMAK700 [11], a pSC101 plasmid with a temperature-sensitive replicon, were digested with *Nae*I, releasing a 9541 bp *tnsABCD *containing fragment and a 2416 bp pSC101 origin-containing fragment, respectively. These two fragments were ligated together to create pGRG3, which contains the *bla *gene encoding β-lactamase, the *araC *gene and the P_BAD_-*tnsABCD *genes, and the temperature-sensitive pSC101 origin of replication. Kan^R ^and Chl^R ^mini-Tn*7*s were inserted into pGRG3 using *red*-*gam *recombination as follows: The primers ATCATGGCAATTCTGGAAGAAATAGCGCTTTCAGCCtgtgggcggacaaaatagttgggaactggga and CATGAGCAGATCCTCTACGCCGGACGCATCGTGGCCtgtgggcggacaataaagtcttaaactgaa were used to amplify the Kan^R ^mini-Tn*7 *from pGPS1.1 and the Chl^R ^mini-Tn*7 *from pGPS2.1 (both from New England Biolabs). The lower case letters are complementary to Tn*7 *and the upper case letters are homologous to sequences in pGRG3. The resulting PCR products were transformed into DH5α carrying the plasmid pTP223 [23], which expresses the phage λ *red *recombinase products, and Kan^R ^or Chl^R ^recombinants were selected. The resulting Kan^R ^and Chl^R ^mini-Tn*7 *containing plasmids were named pGRG8 and pGRG6. Both Tn*7 *inserts in the plasmids were sequenced to ensure that no mutations were inserted by PCR. Any mutations were corrected to the wildtype sequence by subcloning from samples that did not contain those mutations. pGRG17 was created by digesting pGRG8 with *Dra*III and *Eag*I to remove the kanamycin resistance gene. This was replaced with the annealed oligonucleotides GGCCGTGGCGCGCCTCCTAGGTGCTCGAGTGGCGGCCGCTATTGAGGGATCTGATTAATTAAAAC and TTAATTAATCAGATCCCTCAATAGCGGCCGCCACTCGAGCACCTAGGAGGCGCGCCAC to create a multiple cloning site with the following unique restriction sites: *Avr*II, *Not*I, *Pac*I, and *Xho*I.

To provide conjugation as an alternative way to introduce these plasmids into bacterial strains, we cloned the *oriT *site from the RP4 plasmid sequences in the strain SM10 [24]. *oriT *was amplified by PCR using primers GGCGCCGGCCAGCCTCGCAGAGCA and GGCGCCGGGCAGGATAGGTGAAGT, and cloned into pCR2.1-topo using the Topo TA cloning kit (Invitrogen). The sequence and transfer activity was confirmed, then the *oriT *sequence was moved by moving the 113 bp *Sfo*I fragment into the *Nae*I site (resulting in the plasmid in parentheses) of pGRG6 (pGRG19), pGRG8 (pGRG20), and pGRG17 (pGRG25). pGRG36 is identical to pGRG25, except that it contains a *Sma*I site in the multiple cloning site. Both pGRG25 and pGRG36 sequences can be downloaded at GenBank using NCBI accession # DQ460223. The ability of the delivery plasmids to be transferred by conjugation was confirmed using pGRG25-containing SM10 as donor in a standard conjugation assay at 32°C [25].

To facilitate the cloning of large fragments into the delivery vehicle we cloned the RfC.1 element from the Gateway cloning system (Invitrogen) into the *Sma*I site of pGRG36, yielding pGRG37. This allows *in vitro *movement of transgenes cloned in Gateway cloning vectors into pGRG37, bypassing the need for standard cloning techniques [26].

### Plasmid and strain handling

Plasmids and strains were prepared as described [27] with the exception that transposition plasmids were grown in LB medium with ampicillin and 0.1% glucose, to repress transposition, and plasmids were prepared using Qiagen very low-copy plasmid protocols.

### Transformation and transposition protocol

*E. coli *electro-competent cells were prepared normally, as described in [27], with the exception that they were grown in Luria-Bertani (LB) medium with 0.1% glucose to repress expression of the transposase during recovery. Transformants were selected on LB + 100 μg/mL ampicillin at 32°C and streaked out once, then grown overnight in LB at 32°C, with no drugs present. Dilutions of this culture were plated on LB + 30 μg/mL chloramphenicol (pGRG19) or on LB alone (pGRG25). Colonies were streaked at least once at 42°C to ensure plasmid loss. Transposition was verified by absence of the ampicillin resistance marker and by PCR using the primers 5'GATGCTGGTGGCGAAGCTGT and 5'GATGACGGTTTGTCACATGGA, which flank the *attTn7 *site.

## Authors' contributions

GJM conceived of and constructed the system, designed and conducted experiments and wrote the manuscript. NLC designed experiments and edited the manuscript.

## Figures and Tables

**Table 1:** Frequency of transgene insertion at *E. coli attTn7 *in the absence of selection. For each experiment shown three independent electrotransformations of pGRG25, which carries the multiple cloning site as the transgene, into strain MG1655 were performed. One single colony from each transformation was streaked out once at 32°C on LB with ampicillin, and then a culture of each colony was grown overnight in LB at 32°C. Appropriate dilutions were then plated on LB at 42°C. Ten colonies from each culture were examined by PCR to determine if they carried an insertion at the attachment site. The values shown are the average frequency from 3 cultures with 10 colonies examined per culture (*i.e*., a total of 30 colonies examined per experiment). The final value shows a mean average of all 6 experiments and the standard error of the mean (SEM).

Experiment	Frequency of colonies carrying Tn*7 *at attachment site
1	0.83
2	0.70
3	0.63
4	0.70
5	0.90
6	0.97
Average ± SEM	0.79 ± 0.05

**Table 2:** Frequency of transgene insertion into sites other than *attTn7*. For each experiment shown, three independent electrotransformations of pGRG19 (carrying a Chl^R^ transgene) into MG1655 and MG1655 *attTn7*::Tn*7kan *were done. The presence of the Tn*7 *at *attTn7 *prevents insertion events there and allows measurement of the frequency at which Tn*7 *can transpose to other sites as discussed in the text [13]. One single colony from each transformation was streaked out once at 32°C on LB with ampicillin. From this, a culture was grown overnight in LB at 32°C, and appropriate dilutions were plated at 42°C on LB and LB with chloramphenicol. Values shown are the frequency of Chl^R^

Strain	Experiment	Frequency of Chl^R ^cells	Average ± SEM
	1	7.0 × 10^-1^	
MG1655	2	7.2 × 10^-1^	(7.9 ± 0.8) × 10^-1^
	3	9.4 × 10^-1^	

	1	6.8 × 10^-5^	
MG1655 *attTn7*::Tn*7*	2	4.6 × 10^-5^	(5.5 ± 0.7) × 10^-5^
	3	5.2 × 10^-5^	
